# In this month’s Bulletin

**DOI:** 10.2471/BLT.22.000622

**Published:** 2022-06-01

In the editorial section, Ryan McBain et al. (358) introduce an initiative to track HIV resource allocation and costs. Jane Barratt (359) argues for specific measures to improve vaccination coverage in older adults.

Gary Humphreys (362–363) reports on continued resolve in the effort to eradicate polio and talks to John Nkengasong (364–365) about the establishment of the Africa Centers for Disease Control.

## Bosnia and Herzegovina, Brazil, Bulgaria, Cameroon, China, Cuba, Egypt, Ethiopia, India, Islamic Republic of Iran, Iraq, Mexico, Russian Federation, Serbia, South Africa, Thailand, Turkey, Uganda, West Bank and Gaza Strip, Zambia

### Chronic occupational stress in primary health-care providers

Tanya Wright et al. (385–401) review the evidence for factors associated with burnout.

## India

### Preventing influenza, pneumococcal disease, typhoid and hepatitis B

Ali Abbas Rizvia & Abhishek Singh (375–384) explore predictors of vaccine uptake in older adults.

## Egypt

### Continuing professional development 

Mohamed Reda Bassiouny and Azza R Elhadidy (402–408) describe foundations of an effective system.

## Global

### Effective diagnosis of SARS-CoV-2 infections 

Miguel Angel Garcia-Bereguiain et al. (411–412) argue for more stringent regulation of test kits. 

## Preventing abuse in the health-care sector

Tamim Alsuliman et al. (409–410) make the case that formal guidelines are needed.

### Maintaining global influenza surveillance

Margaret McCarron et al. (366–374) examine laboratory network performance in the context of reduced funding. 

